# Longitudinal Changes in Corneal Thickness over 8 Years: Findings from the National Institute for Longevity Sciences–Longitudinal Study of Aging Population-Based Cohort Study in Japan

**DOI:** 10.1016/j.xops.2025.100860

**Published:** 2025-06-19

**Authors:** Hideki Fukuoka, Chikako Tange, Fujiko Ando, Hiroshi Shimokata, Yukiko Nishita, Rei Otsuka

**Affiliations:** 1 Department of Ophthalmology, Kyoto Prefectural University of Medicine, Kyoto, Japan; 2 Department of Epidemiology of Aging, Research Institute, National Center for Geriatrics and Gerontology, Obu, Japan; 3 Faculty of Health and Medical Science, Aichi Shukutoku University, Nagakute, Japan; 4 Graduate School of Nutritional Sciences, Nagoya University of Arts and Sciences, Nisshin, Japan

**Keywords:** Central corneal thickness, Keratocytes, Age-related changes, Longitudinal study, Community dweller

## Abstract

**Purpose:**

To evaluate age-related changes in central corneal thickness (CCT) and investigate its relationship with other ocular parameters in community-dwelling Japanese adults through an 8-year longitudinal analysis.

**Design:**

A population-based, prospective longitudinal cohort study with baseline measurements from 1997 to 2000 and follow-up from 2006 to 2008.

**Subjects:**

A total of 631 community-dwelling Japanese adults aged 40 to 79 years (mean age: 55.7 ± 9.7 years) were enrolled from the National Institute for Longevity Sciences–Longitudinal Study of Aging. We excluded participants with corneal pathologies, contact lens use, glaucoma medication, or missing endothelial cell density measurements.

**Methods:**

Central corneal thickness was measured using calibrated specular microscopy (SP-2000; Topcon Corporation) at 2 time points approximately 8 years apart. Secondary measurements included corneal endothelial cell density, coefficient of variation in cell size, and corneal curvature. Mixed-effects models analyzed CCT changes, adjusting for sex, season, corneal endothelial cell density, and systemic health factors.

**Main Outcome Measures:**

Age-related changes in CCT, annual rate of CCT change across different age decades, and correlations between CCT changes and ocular/systemic parameters.

**Results:**

At baseline, adjusted CCT measurements were 520.2 ± 2.1 (standard error [SE]) μm, 514.1 ± 2.2 μm, 518.0 ± 2.5 μm, and 514.7 ± 3.7 μm for participants in their 40s, 50s, 60s, and 70s, respectively. Longitudinal analysis revealed a significant increase in CCT over time across all age groups (β = 0.7; SE = 0.1; *P* < 0.001). The annual CCT increase showed age-dependent slowing: 0.68 ± 0.08 μm for 40s, 0.62 ± 0.08 μm for 50s, 0.46 ± 0.09 μm for 60s, and 0.20 ± 0.14 μm for 70s with a statistically significant difference between 40s and 70s groups (β = −0.5; SE = 0.2' *P* = 0.003).

**Conclusions:**

This longitudinal analysis demonstrates that CCT increases over time in all age groups, with the rate of increase significantly slowing in older age groups. These findings contrast with previous cross-sectional studies suggesting CCT decreases with age, emphasizing the importance of longitudinal observations. These results have important implications for glaucoma diagnosis and refractive surgery safety evaluations in aging populations.

**Financial Disclosure(s):**

The author(s) have no proprietary or commercial interest in any materials discussed in this article.

Central corneal thickness (CCT) measurement is fundamental in ophthalmology, providing crucial information for both clinical diagnosis and surgical planning. The cornea's complex structure, comprising epithelial cells, Bowman layer, stroma, Descemet membrane, and endothelial cells, forms the transparent front part of the eye that enables light transmission and vision focusing.^1^ Commonly, CCT is measured in micrometers (μm) via the use of minimally invasive methods such as ultrasound pachymetry, noncontact specular microscopy,^2^ anterior segment OCT,^3^ and others.

Although it can vary among individuals, the normal CCT measurement value ranges between 500 and 600 μm, and it is of particular importance in ocular disorders such as glaucoma, where corneal thickness can have an impact on intraocular pressure (IOP) measurements—a critical parameter in the diagnosis and monitoring of glaucoma. Moreover, it has been reported that in subjects with a thinner CCT, there is a heightened risk for the development of open-angle glaucoma.^4^ Hence, CCT measurements are now included as a standard procedure in the evaluation of glaucoma.

In addition, the CCT is a critical parameter in refractive surgery, such as laser in situ keratomileusis, photorefractive keratectomy, and others used for the treatment of corneal disorders, as corneal reshaping procedures to correct vision can lead to corneal thinning and alterations in corneal biomechanical properties and IOP.^5^ To assess whether a patient's corneal stromal bed is thick enough to safely undergo a refractive surgery procedure without risking complications such as ectasia (i.e., corneal thinning and protrusion), preoperative measurement of CCT is vital because it plays a critical role in determining appropriate surgical candidacy and optimizing treatment outcomes.

The aim of this study was to investigate longitudinal changes in CCT among Japanese adults over an 8-year period while examining its relationship with other ocular parameters and systemic health factors. This study is unique in providing long-term follow-up data from an East Asian population, utilizing consistent measurement protocols and comprehensive health assessments. Understanding these changes is crucial for improving our approach to age-related eye care and surgical planning in Asian populations.

## Methods

### Study Design and Participants

Study participants were selected from the first (1997–2000) and fifth (2006–2008) waves of the National Institute for Longevity Sciences–Longitudinal Study of Aging because these specific waves were selected because CCT measurements were only obtained at these 2 timepoints.

**Figure 1:**
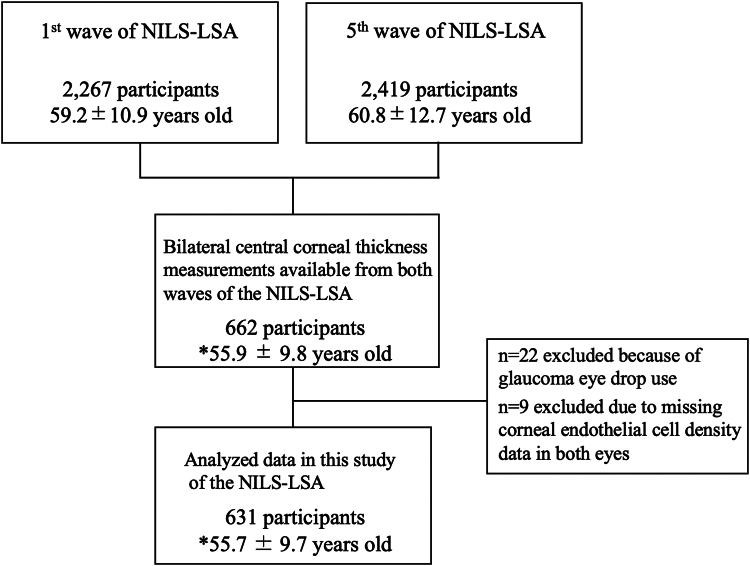
Flowchart of participant selection and exclusion criteria. ∗Age values indicate the age of participants at the first wave. NILS = National Institute for Longevity Sciences; LSA = Longitudinal Study of Aging.

The National Institute for Longevity Sciences–Longitudinal Study of Aging is a longitudinal, dynamic cohort study that includes medical, physiological, nutritional, and psychological assessments. For the first wave, a total of 2267 male and female subjects (mean age: 59.2 ± 10.9 years) were randomly selected using stratified random sampling by sex and age from Obu City and Higashiura Town, municipalities geographically located in the central region of Japan. Of those 2267 first-wave participants, a total of 1262 eyes of 631 participants (mean age: 55.7 ± 9.7 years at wave 1) who were measured at both the first wave and fifth wave, thus representing the baseline and follow-up, respectively, were selected for this present longitudinal analysis after excluding participants with corneal pathologies, history of corneal surgery, contact lens use, no data on either the left or right eye at baseline and follow-up, or glaucoma eye-drop medications used (n = 22). Additionally, 9 participants with no corneal endothelial cell density data in either eye were excluded (Fig 1). No prior sample size calculation was performed. The sample size was determined by the number of participants who had complete CCT measurements at both timepoints. For compilation and presentation of epidemiological data in a table, the right eye was selected as the representative study eye, consistent with conventional methodology applied in ophthalmology research. For statistical analysis, as detailed in the Statistical Analysis section, data from both eyes were included in the mixed-effects models with random intercepts to adjust for intereye correlation within individuals. The protocols of this study were approved by the Ethics Committee of the National Center for Geriatrics and Gerontology, Obu City, Aichi, Japan, and in accordance with the tenets set forth in the Declaration of Helsinki, prior written informed consent was obtained from all participants.

### Ocular Parameters

Central corneal thickness (μm), corneal endothelial cell density (cells/mm^2^), and coefficient of variation in cell size (%) were all measured via the use of the same model noncontact specular microscope (SP-2000; Topcon Corporation) at both baseline and follow-up. The specular microscope was appropriately calibrated according to the manufacturer’s guidelines. Radius of corneal curvature 1 (mm), radius of corneal curvature 2 (mm), and corneal astigmatism (diopters): the same model automated refractometer/keratometer (ARK-700A; Nidek Co., Inc.) was used at both timepoints. As with the specular microscope, the refractometer/keratometer was appropriately calibrated based on the standard procedures and manufacturer’s guidelines. To ensure measurement reliability, all CCT measurements were carefully sequenced to be performed before any potentially invasive procedures that might influence corneal parameters. Using the same appropriately calibrated devices helped ensure the reliability and accuracy of the measurements over time in this longitudinal analysis. For the analysis of each participant, 3 consecutive readings were taken for both eyes and then averaged. All ocular measurements were conducted during the daytime (09:00–15:00) across all participants.

### Other Variables

At baseline, additional assessments were performed, including body weight and height, blood pressure (systolic and diastolic), tobacco use (smoking), and self-reported history of stroke, ischemic heart disease, hypertension, dyslipidemia, diabetes mellitus, corneal endothelial cell density, and season of examination. Corneal endothelial cell density was measured in both eyes when available; for participants with data from both eyes, the average value was used, whereas for those with data from only 1 eye, that single measurement was used. The seasons were categorized as Spring (March–May), Summer (June–August), Autumn (September–November), and Winter (December–February) based on the examination date.

### Statistical Analysis

Data processing and analysis were performed using SAS v.9.3 (SAS Institute, Inc.) statistical analysis software. In this study, 4 age groups were used (i.e., subjects in their 40s, 50s, 60s, and 70s) categorized according to their baseline age.

Mixed-effects models were used to evaluate age-related changes in CCT over an 8-year period because these models can appropriately handle variables that are correlated within individuals better than traditional regression analysis. More details on applying mixed-effects models to analyze correlated continuous eye data are published elsewhere.^6^

The model used in this study included fixed terms for the intercept, age groups at baseline (40s, 50s, 60s, and 70s), time in years since baseline (slope), and the interaction of age group × time, and it was adjusted for sex, season, and corneal endothelial cell density. The age group × time interaction tested whether the CCT change over time (slope) varied according to the baseline age groups.

Additionally, random intercepts were included to adjust for intereye correlation within individuals. *P* < 0.05 was set as the level of significance for all analyses. Estimated CCT values were calculated for each decade. The slope represents the rate of CCT change per year in each decade.

We also conducted a supplementary analysis excluding participants who underwent cataract surgery between baseline and follow-up (n = 52) to assess whether surgical intervention influenced the observed trends in CCT changes. This analysis used the same model structure, which included fixed terms for the intercept, age groups at baseline (40s, 50s, 60s, and 70s), time in years since baseline (slope), and the interaction of age group × time, and was adjusted for sex, season, and corneal endothelial cell density.

## Results

The demographic characteristics and right eye measurements of the enrolled participants at the baseline of National Institute for Longevity Sciences–Longitudinal Study of Aging are shown in Table 1.

**Table 1:** Characteristics of the Participants (n = 631)

Variables	Unit	Value
Age	Years old	55.7 ± 9.7
Sex (female)		50.2%
Number of participants in their 40s (male/female)		102/107
50s		100/99
60s		83/73
70s		29/38
Body weight	kg	58.2 ± 9.9
Body height	cm	159.2 ± 8.5
Systolic blood pressure	mmHg	122.5 ± 17.9
Diastolic blood pressure	mmHg	75.3 ± 10.4
Smoking		42.4%
Stroke		1.3%
Ischemic cardiac disease		9.2%
Hypertension		21.1%
Hyperlipidemia		17.3%
Diabetes mellitus		5.3%
Central corneal thickness	μm	514.8 ± 33.2
Radius of corneal curvature 1	mm	7.71 ± 0.27
Radius of corneal curvature 2	mm	7.57 ± 0.26
Corneal astigmatism	diopters	−0.84 ± 0.65
Corneal endothelial cell density	cells/mm^2^	2520 ± 374
Mean conical endothelial cell size	μm^2^	406.5 ± 72.7
Coefficient of variation of corneal endothelial cell size	%	41.7 ± 17.4

Note: Data presented as available for each measure with minor missingness documented in systolic (1) and diastolic (1) blood pressure, smoking status (1), hypertension (1), hyperlipidemia (2), diabetes (2), corneal curvature measures (3), and corneal endothelial metrics (16).

The mean time period between baseline (first wave) and follow-up (fifth wave) was 8.22 ± 0.42 years.

### Cross-Sectional Analysis of Age and CCT at Baseline

**Figure 2:**
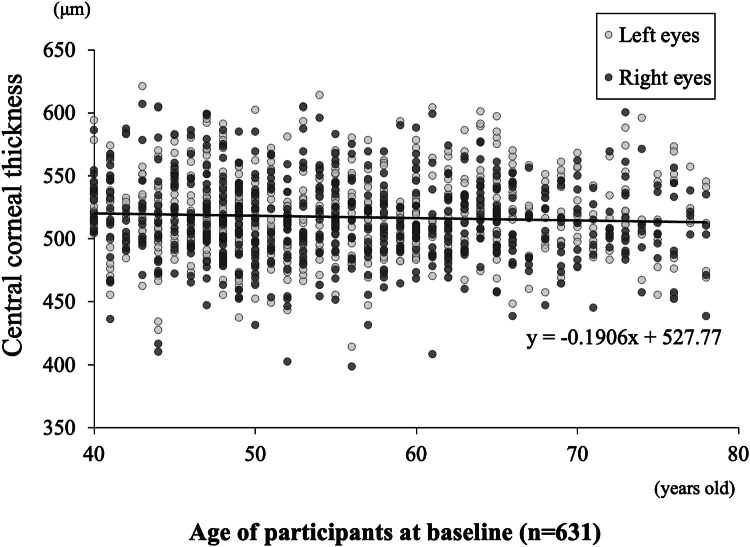
The plot between age and CCT at the first wave. Black dots indicate right eyes, and gray dots indicate left eyes. Cross-sectional analysis revealed a trend toward thinner CCT with increasing age (y = −0.19x + 527.77; y = CCT; x = age at the first wave) (n = 631). CCT = central corneal thickness.

To provide a clear visual representation, a scatter plot illustrating the relationship between age and CCT at baseline was used, and our findings from this cross-sectional analysis showed a trend toward thinner CCT with increasing age in both eyes (y = −0.19x + 527.77; y = CCT; x = age at first wave) (Fig 2). This cross-sectional analysis reflects differences between age groups at a single point in time, which we later contrast with our longitudinal findings that track changes within individuals over the 8-year period.

### Longitudinal Analysis of Age-Related Changes in CCT

**Figure 3:**
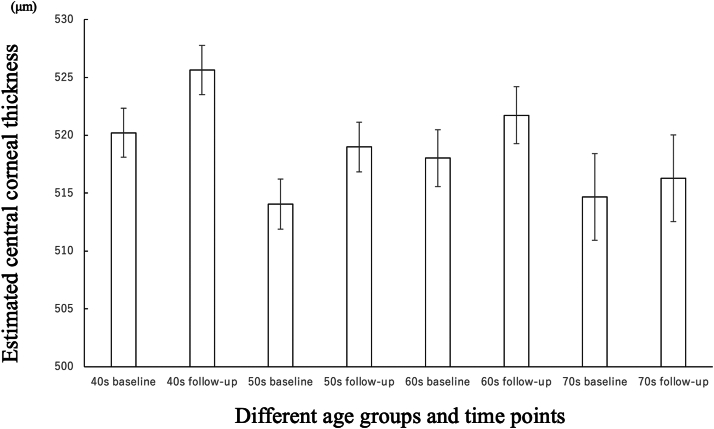
Changes in adjusted CCT over 8 years, stratified by age group at the first wave. Longitudinal changes in central corneal thickness over 8 years. Estimated CCT and error ranges after adjustment for sex, season, and corneal endothelial cell density for each age group at baseline and after an average observation period of 8.22 years (n = 631). CCT = central corneal thickness.

Table 2 shows the results of mixed-effects models to evaluate age-related changes in CCT. No significant differences in CCT were found between the age groups at baseline. The effect of time was statistically significant (β = 0.7; standard error [SE] = 0.1; *P* < 0.0001). Regarding the interaction between age group and time, the difference in CCT change slope between the 40s groups and the 70s groups was statistically significant (β = −0.5; SE = 0.2; *P* = 0.003) over 8 years. The interaction effects between other age groups over time were not statistically significant. The estimated CCT in the 40s, 50s, 60s, and 70s at baseline was 520.2 ± 2.1 (SE), 514.1 ± 2.2 (SE), 518.0 ± 2.5 (SE), and 514.7 ± 3.7 (SE) μm after adjustment for sex, season, and corneal endothelial cell density, respectively. After an average interval at follow-up, the estimated CCT in the 40s, 50s, 60s, and 70s groups was 525.7 ± 2.1 (SE), 519.0 ± 2.2 (SE), 521.7 ± 2.5 (SE), and 516.3 ± 3.7 (SE) μm after adjusting for sex, season, and corneal endothelial cell density, respectively. Figure 3 shows the estimated CCT for each age group at baseline and follow-up.

**Table 2:** Mixed-Effects Model of Fixed Effects Adjusted for Sex, Seasons, and Corneal Endothelial Cell Density (n = 631)

Effect	β	SE	*P*
Intercept	499.3	9.6	<0.001
Age group 40s		Reference	
Age group 50s	−6.2	3.0	0.043
Age group 60s	−2.2	3.3	0.502
Age group 70s	−5.5	4.3	0.200
Time	0.7	0.1	<0.001
Time∗age group 40s		Reference	
Time∗age group 50s	−0.1	0.1	0.569
Time∗age group 60s	−0.2	0.1	0.070
Time∗age group 70s	−0.5	0.2	0.003

SE = standard error.

“^∗^” Indicates interaction term.

**Figure 4:**
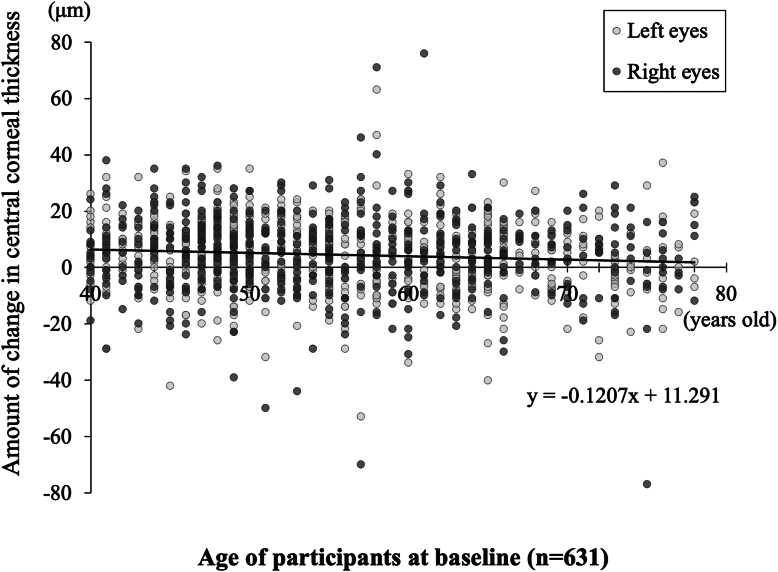
Plot between age and delta (change) in CCT. Black dots indicate right eyes, and gray dots indicate left eyes. Longitudinal analysis revealed a decreasing rate of change in CCT with increasing age over a period of approximately 8 years (y = −0.12x + 11.29; y = Δ CCT; x = age at the first wave) (n = 631). CCT = central corneal thickness.

The positive slope of CCT per year in the 40s, 50s, 60s, and 70s groups was 0.68 ± 0.08 μm (SE), 0.62 ± 0.08 μm (SE), 0.46 ± 0.09 μm (SE), and 0.20 ± 0.14 μm (SE), respectively. In our study, the percentage increase in CCT between that at the baseline and at the 8-year follow-up examination in the 40s, 50s, 60s, and 70s groups was approximately 1.03%, 0.96%, 0.72%, and 0.31%, respectively, with the slope of change decreasing with older age. Furthermore, the scatter plot used to visually examine the relationship between age and amount of change in CCT revealed a decreasing rate of change in CCT with increasing age over a period of approximately 8 years in longitudinal analysis (y = −0.12x + 11.29; y = Δ CCT; x = age at the baseline) (Fig 4).

### Supplementary Analysis Excluding Participants with Cataract Surgery

In our supplementary analysis, we excluded 52 participants who underwent cataract surgery between baseline and follow-up. The results of this analysis were largely consistent with our primary findings. The effect of time remained statistically significant (β = 0.7; SE = 0.1; *P* < 0.001), and the interaction between the 40s and 70s age groups over time continued to be significant (β = −0.6; SE = 0.2; *P* = 0.001). Interestingly, in this supplementary analysis, we also detected a significant interaction between the 40s and 60s age groups (β = −0.3; SE = 0.1; *P* = 0.027), which was not observed in our primary analysis. These findings suggest that although cataract surgery did not fundamentally alter the overall age-related patterns of CCT change in our cohort, its exclusion revealed additional age-specific differences in CCT change rates (Supplemental Table 1, available at www.ophthalmologyscience.org, Supplemental Figure 1, available at www.ophthalmologyscience.org).

## Discussion

The aim of this study was to investigate changes in CCT over the life course in those ≥40 years through a longitudinal analysis. Our findings revealed a significant increase in CCT with time after adjusting for sex, season, and corneal endothelial cell density, with the rate of increase declining in older age groups. These findings remained consistent even when excluding patients who underwent cataract surgery between baseline and follow-up.

The findings of this study demonstrate a significant distinction between cross-sectional and longitudinal CCT analyses. Although cross-sectional data indicated a correlation between thinner CCT and increasing age, longitudinal data revealed that CCT increases over time in all age groups. This discrepancy is likely attributable to cohort effects, whereby distinct generations encounter disparate early-life nutritional and socioeconomic conditions, a phenomenon that is particularly salient in the context of 20th century Japan. This underscores the value of longitudinal studies in providing more precise insights into age-related corneal changes when compared with cross-sectional analyses alone.

Five previous longitudinal studies have reported age-related change and rate of change in CCT. In the Ocular Hypertension Treatment Study, the overall decrease in CCT in 1191 subjects was 2.7 μm after an average follow-up duration of 3.8 years, regardless of whether participants were on IOP-lowering medication or not.^7^ Moreover, the decrease in CCT observed in the subset of untreated subjects was 3.5 μm. The Chennai Glaucoma Follow-up Study reported a 3.46 μm decrease after a 3-year follow-up of 196 glaucomatous patients.^8^ Analysis of CCT measurements obtained in 2509 healthy Iranians who participated in the Shahroud Cohort Eye Study revealed that the last measurement was 1.5 μm significantly thinner than the first one taken 5 years earlier. In that study, the proportions of eyes that showed negative, positive, and no change in CCT were 56.6%, 41.4%, and 2.0%, respectively.^9^ Weizer et al retrospectively analyzed 2 sets of CCT measurements collected 8.3 years apart in 39 subjects, including 12 with normal eye examinations, 21 with various types of glaucoma, 1 glaucoma suspect, and 5 with ocular hypertension. Their results showed significant decreases in CCT of 17 μm in right eyes and 23 μm in left eyes over the follow-up period.^10^ In the Tema Eye Survey, Mwanza et al conducted a longitudinal study of 758 normal and 58 glaucomatous eyes over an average of 8.4 years. They found significant overall decreases of 8.9 μm in the right eye and 9.8 μm in the left eye, with glaucomatous eyes showing significantly greater decreases (right eye: −14.1 vs. −8.6 μm, left eye: −14.5 vs. −9.5 μm) compared with normal eyes.^11^

Although these longitudinal studies demonstrate temporal changes in CCT, multiple cross-sectional studies have provided additional insights into racial variations in CCT^12^, ^13^, ^14^, ^15^, ^16^, ^17^, ^18^, ^19^, ^20^ (e.g., Latinos vs. non-Latino whites,^12^ African Americans vs. non-Hispanic Whites and Hispanics^13^ etc) and sex^13^^,^^21^ and is positively associated with IOP.^5^ Regarding differences in CCT with age (i.e., age group), most cross-sectional studies reported no significant differences^22^ or that corneal thickness became thinner with increasing age.^13^^,^^14^ In a detailed analysis within the present study, the results of examining mean corneal thickness for each age group showed that there seemed to be a decrease in thickness with increasing age, similar to the findings in previous reports; however, the differences between age groups were not statistically significant in our data.

Our study is distinct from previous investigations in several important ways. First, we adjusted for corneal endothelial cell density, which is known to decline with age and could potentially influence CCT. The inclusion in our models did not substantially alter the age-related patterns of CCT change, suggesting that the observed increase in CCT over time may be attributed to structural changes in other corneal layers beyond the endothelium alone. Additionally, our analysis confirmed that seasonal variations did not significantly influence CCT measurements, addressing concerns about potential confounding due to environmental factors.

Furthermore, our supplementary analysis excluding participants who underwent cataract surgery between baseline and follow-up (52 participants, representing approximately 8.2% of the total cohort) yielded results consistent with our primary findings. This consistency may be partly attributable to the relatively small proportion of participants who underwent cataract surgery during the follow-up period. Nevertheless, this important verification addresses concerns about potential surgical influences on corneal parameters and further strengthens the validity of our conclusions regarding age-related CCT changes.

As for possible mechanisms in the change of CCT, it is important to note that the cornea is made up of epithelium, the Bowman layer, the stroma, the Descemet membrane, and endothelium and that the CCT is the sum of the thicknesses of those layers. Thus, it was not clear which layers had changed. The Descemet membrane is located between the corneal stroma and the corneal endothelial cells, and it is considered to be the basement membrane of the endothelial cells.^1^ The Descemet membrane demonstrates a significant increase in thickness with advancing age. In infants, it is only 3 to 4 μm thick, approximately 1 of 3 thicknesses found in adults (10-12 μm).^23^ This age-related thickening is attributed to additional collagen production by the corneal endothelium. Similarly, corneal stroma may be remodeled or regenerated by corneal epithelial cells and stromal keratocytes.^24^

As stated above, the rate of increase in CCT over the 8-year study period declined with advancing age, from 1.03% in the 40s age group to 0.31% in the 70s age group. It is already known that aging in humans reduces tissue metabolism and regenerative capacity and that it is influenced by many factors, including individual genetic background, lifestyle, environment, and health status.^25^^,^^26^ Moreover, the general trend is a rapid decline in muscle mass and strength from the age of 60 years^27^ and in bone density and nerve function from the age of 70 years.^28^ It is thought that this may be due to a decline in collagen production^29^ and other regenerative forces in older age groups, as mentioned above, and that the rate of increase is less pronounced in people in their 70s than in other age groups.

Several studies have suggested relationships between CCT and various conditions, including diabetes, metabolic syndrome, high body mass index, and chronic kidney disease. However, the evidence is inconclusive regarding a relationship between these conditions and CCT, due to various studies reporting conflicting results. For example, although some studies have reported associations between a thinner CCT and diabetes or hyperglycemia,^30^ others have found no significant relationships.^31^ Thus, more research controlling for potential confounding factors is needed to clarify whether CCT is an independent risk factor for these conditions or if positive associations can be explained by other linked factors.

Some potential limitations of the present study are the enrollment of exclusively Japanese individuals from the general population. However, this gave us the advantage of a more focused analysis within a single racial/ethnic group, rather than including multiple racial/ethnic groups. It is already known from several publications that prostaglandins as glaucoma eye drops significantly decreased corneal epithelium, corneal stroma, and total CCT after eye drop application.^32^ In this study, we investigated whether or not eye drops were used, as well as the types of eye drops administered when used. Whenever possible, we excluded patients using prostaglandin/beta blocker or other glaucoma drops. In addition, we did not adjust for potential confounding factors such as diabetes, metabolic syndrome, and body mass index in our analysis of CCT over time.

In conclusion, our investigation of CCT in the Japanese population identified important variations in CCT in age. The longitudinal study showed an increase in CCT over time after adjusting for corneal endothelial cell density and seasonal variations, and this finding remained consistent even when excluding patients who underwent cataract surgery. Furthermore, the rate of change in CCT appears to decrease with age. These findings may have important implications for the clinical understanding of eye health, particularly in the context of glaucoma evaluation and refractive surgery. However, further research is needed to fully elucidate the exact mechanisms and environmental factors influencing these variations in CCT.

## References

[1] Leung E.W., Rife L., Smith R.E., Kay E.D.P. (2000). Extracellular matrix components in retrocorneal fibrous membrane in comparison to corneal endothelium and Descemet's membrane. Mol Vis.

[2] Tao A., Chen Z., Shao Y. (2013). Phacoemulsification induced transient swelling of corneal Descemet's Endothelium Complex imaged with ultra-high resolution optical coherence tomography. PLoS One.

[3] Wang J., Abou Shousha M., Perez V.L. (2011). Ultra-high resolution optical coherence tomography for imaging the anterior segment of the eye. Ophthalmic Surg Lasers Imaging.

[4] Gordon M.O., Beiser J.A., Brandt J.D. (2002). The Ocular Hypertension Treatment Study: baseline factors that predict the onset of primary open-angle glaucoma. Arch Ophthalmol.

[5] Schallhorn J.M., Schallhorn S.C., Ou Y. (2015). Factors that influence intraocular pressure changes after myopic and hyperopic lasik and photorefractive keratectomy: a large population study. Ophthalmology.

[6] Ying G.S., Maguire M.G., Glynn R.J., Rosner B. (2021). Tutorial on biostatistics: longitudinal analysis of correlated continuous eye data. Ophthalmic Epidemiol.

[7] Brandt J.D., Gordon M.O., Beiser J.A. (2008). Changes in central corneal thickness over time. The ocular hypertension treatment study. Ophthalmology.

[8] Choudhari N.S., George R., Sathyamangalam R.V. (2013). Long-term change in central corneal thickness from a glaucoma perspective. Indian J Ophthalmol.

[9] Hashemi H., Asgari S., Emamian M.H. (2016). Five year changes in central and peripheral corneal thickness: the Shahroud Eye Cohort Study. Contact Lens Anterior Eye.

[10] Weizer J.S., Stinnett S.S., Herndon L.W. (2006). Longitudinal changes in central corneal thickness and their relation to glaucoma status: an 8 year follow up study. Br J Ophthalmol.

[11] Mwanza J.C., Tulenko S.E., Budenz D.L. (2018). Longitudinal change in central corneal thickness in the Tema eye Survey. Am J Ophthalmol.

[12] Hahn S., Azen S., Ying-Lai M. (2003). Central corneal thickness in Latinos. Investig Ophthalmol Vis Sci.

[13] Aghaian E., Choe J.E., Lin S., Stamper R.L. (2004). Central corneal thickness of Caucasians, Chinese, Hispanics, Filipinos, African Americans, and Japanese in a glaucoma clinic. Ophthalmology.

[14] Lee E.S., Kim C.Y., Ha S.J. (2007). Central corneal thickness of Korean patients with glaucoma. Ophthalmology.

[15] Kniestedt C., Lin S., Choe J. (2006). Correlation between intraocular pressure, central corneal thickness, stage of glaucoma, and demographic patient data: prospective analysis of biophysical parameters in tertiary glaucoma practice populations. J Glaucoma.

[16] Chua J., Tham Y.C., Liao J. (2014). Ethnic differences of intraocular pressure and central corneal thickness: the Singapore epidemiology of eye diseases study. Ophthalmology.

[17] Eysteinsson T., Jonasson F., Sasaki H. (2002). Central corneal thickness, radius of the corneal curvature and intraocular pressure in normal subjects using non-contact techniques: reykjavik eye study. Acta Ophthalmol Scand.

[18] La Rosa F.A., Gross R.L., Orengo-Nania S. (2001). Central corneal thickness of caucasians and african americans in glaucomatous and nonglaucomatous populations. Arch Ophthalmol.

[19] Nemesure B., Wu S.Y., Hennis A., Leske M.C. (2003). Corneal thickness and intraocular pressure in the Barbados Eye Studies. Arch Ophthalmol.

[20] Pakravan M., Javadi M.A., Yazdani S. (2017). Distribution of intraocular pressure, central corneal thickness and vertical cup-to-disc ratio in a healthy Iranian population: the Yazd Eye Study. Acta Ophthalmol.

[21] Brandt J.D., Beiser J.A., Kass M.A., Gordon M.O. (2001). Central corneal thickness in the ocular hypertension treatment study (OHTS). Ophthalmology.

[22] Shimmyo M., Ross A.J., Moy A., Mostafavi R. (2003). Intraocular pressure, Goldmann applanation tension, corneal thickness, and corneal curvature in Caucasians, Asians, Hispanics, and African Americans. Am J Ophthalmol.

[23] Johnson D.H., Bourne W.M., Campbell R.J. (1982). The ultrastructure of descemet's membrane: I. Changes with age in normal corneas. Arch Ophthalmol.

[24] West-Mays J.A., Dwivedi D.J. (2006). The keratocyte: corneal stromal cell with variable repair phenotypes. Int J Biochem Cell Biol.

[25] López-Otín C., Blasco M.A., Partridge L. (2013). The hallmarks of aging. Cell.

[26] Rando T.A. (2006). Stem cells, ageing and the quest for immortality. Nature.

[27] Keller K., Engelhardt M. (2013). Strength and muscle mass loss with aging process. Age Strength Loss Muscles Ligaments Tendons J.

[28] Vondracek S.F., Linnebur S.A. (2009). Diagnosis and management of osteoporosis in the older senior. Clin Interv Aging.

[29] Varani J., Dame M.K., Rittie L. (2006). Decreased collagen production in chronologically aged skin: roles of age-dependent alteration in fibroblast function and defective mechanical stimulation. Am J Pathol.

[30] Luo X.Y., Dai W., Chee M.L. (2019). Association of diabetes with central corneal thickness among a multiethnic asian population. JAMA Netw Open.

[31] Nishitsuka K., Kawasaki R., Kanno M. (2011). Determinants and risk factors for central corneal thickness in Japanese persons: the Funagata study. Ophthalmic Epidemiol.

[32] Eraslan N., Celikay O. (2023). Effects of topical prostaglandin therapy on corneal layers thickness in primary open-angle glaucoma patients using anterior segment optical coherence tomography. Int Ophthalmol.

