# Supplementary figures and images for: Longitudinal Changes in Corneal Thickness over 8 Years: Findings from the National Institute for Longevity Sciences–Longitudinal Study of Aging Population-Based Cohort Study in Japan
Source: Ophthalmol Sci. 2025 Jun 19;5(6):100860. doi: 10.1016/j.xops.2025.100860 (PMC12304667; doi:10.1016/j.xops.2025.100860)

Supplemental Figure 1.

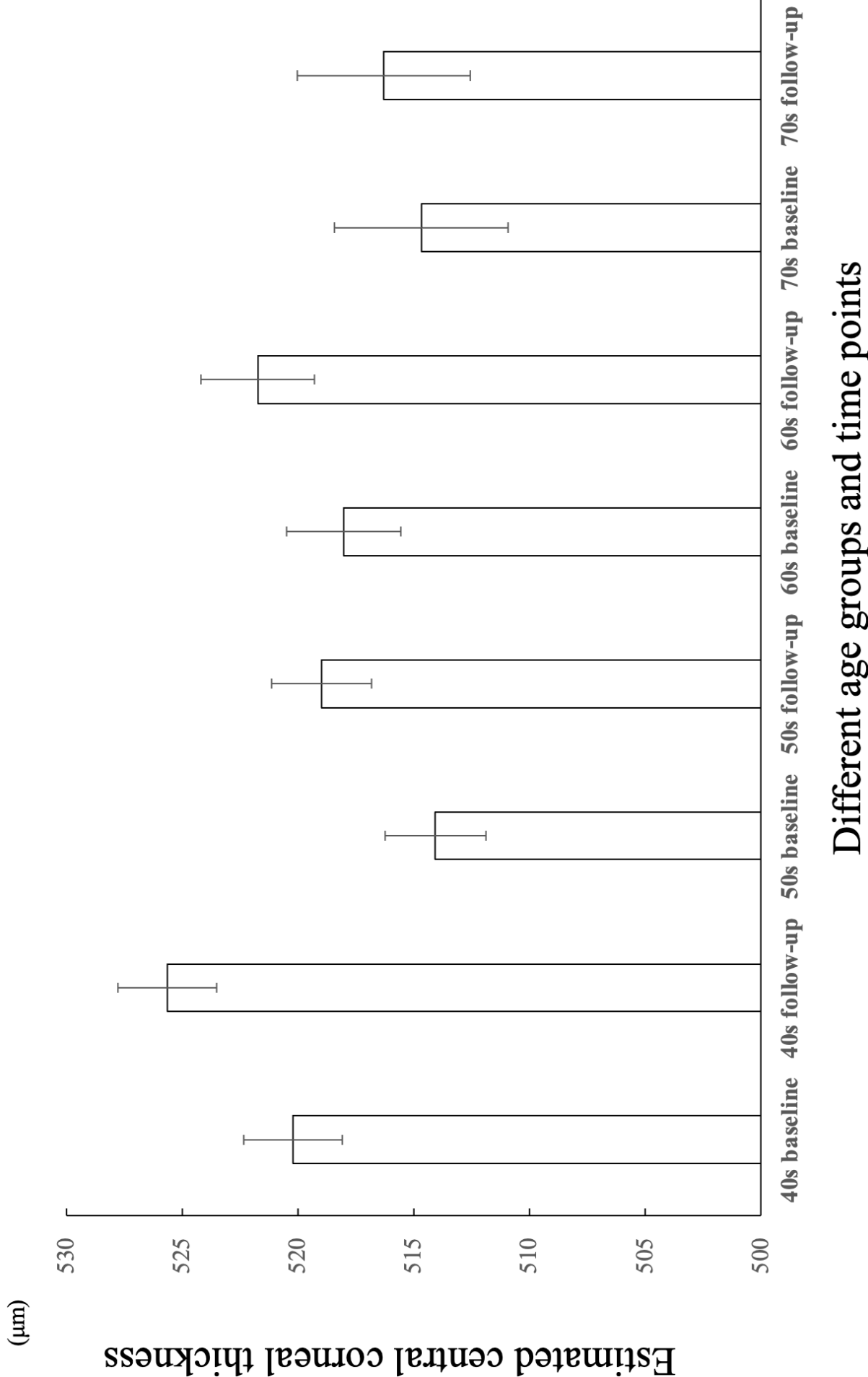

Supplement: Supplemental Figure 1 [file mmc1.pdf]
