# Supplementary material for: Longitudinal Changes in Corneal Thickness over 8 Years: Findings from the National Institute for Longevity Sciences–Longitudinal Study of Aging Population-Based Cohort Study in Japan
Source: Ophthalmol Sci. 2025 Jun 19;5(6):100860. doi: 10.1016/j.xops.2025.100860 (PMC12304667; doi:10.1016/j.xops.2025.100860)
Supplement: Supplemental Table 1 [file mmc2.pdf]

Supplemental Table 1. Mixed-effects Model of Fixed Effects Adjusted for Sex, seasons, and corneal endothelial cell density, excluding 52 participants who underwent cataract surgery between baseline and follow-up (n=579).

Effect	β	SE	p
intercept	494.9	10.1	<.001
age group 40s		ref	
age group 50s	-5.9	3.1	.058
age group 60s	-1.2	3.4	.715
age group 70s	-5.8	5.0	.245
time	0.7	0.1	<.001
time*age group 40s		ref	
time*age group 50s	-0.1	0.1	.409
time*age group 60s	-0.3	0.1	.027
time*age group 70s	-0.6	0.2	.001
